# Managing challenging behaviour in preschool children post-traumatic brain injury with online clinician support: protocol for a pilot study

**DOI:** 10.1186/s40814-017-0140-0

**Published:** 2017-05-31

**Authors:** Kaitlyn Taylor, Cathy Catroppa, Celia Godfrey, Audrey McKinlay, Jennie Ponsford, Jan Matthews, Vicki Anderson

**Affiliations:** 1 Child Neuropsychology, Murdoch Childrens Research Institute, 50 Flemington Rd, Parkville, Victoria 3052 Australia; 2 Psychological Sciences, Monash University, Melbourne, Australia; 3 Psychological Sciences, University of Melbourne, Melbourne, Australia; 4 Psychology Service, Royal Children’s Hospital, Melbourne, Australia; 5 Department of Paediatrics, University of Melbourne, Melbourne, Australia; 6 Parenting Research Centre, Melbourne, Australia

**Keywords:** Paediatric traumatic brain injury, Behaviour, Parenting programme, Intervention

## Abstract

**Background:**

Traumatic brain injury (TBI) in children is associated with a range of poor long-term outcomes, including behavioural disturbances. Parents can experience high levels of stress and injury-related burden, and evidence suggests that distressed parents are less likely to adopt positive parenting styles to manage their child’s behaviour. The ‘Signposts for Building Better Behaviour’ program is a parenting programme that was originally developed to assist parents of children with an intellectual disability in managing their child’s behaviour. More recently, it has been adapted to include a TBI module, to assist parents in managing post-TBI behaviour. However, geographical and financial barriers remain, preventing many parents from accessing the programme in the standard face-to-face modality. This project aims to investigate the feasibility and acceptability of the programme when delivered with clinician support via videoconferencing.

**Methods/design:**

The sample for this feasibility study will be recruited from the Royal Children’s Hospital, Melbourne, and the Victorian Paediatric Rehabilitation Service. Participants will be the parents of a child who sustained a TBI between the ages of 2.0 and 6.11, within the previous 2 years. The parents of 15 children will complete the programme, with clinician support via videoconferencing, while the parents of a further 15 children will form a treatment as usual wait-list control group. Parents complete questionnaires assessing their child’s behaviour, as well as assessing their own mental health, sense of parenting competency, disciplinary style, and family functioning. These will be completed upon enrolment in the study regarding their child’s pre-injury behaviour and then again pre-intervention, immediately post-intervention, and 4 months post-intervention. Parents who complete the intervention will also complete questionnaires assessing their satisfaction with the programme and its delivery. Information will be collected on the feasibility, clinical practicality, and acceptability of the programme when delivered through this medium.

**Discussion:**

This study is the first to investigate the feasibility of delivering post-child TBI behavioural intervention via videoconferencing in Australia. Preliminary findings from this study may support the development of a larger randomised controlled trial. It is hoped that programme delivery through this medium would facilitate better access to the programme, enabling improved long-term outcomes for families.

**Trial registration:**

ANZCTR, ACTRN12616001574437

## Background

### Child behavioural outcomes post-TBI

Paediatric traumatic brain injury (TBI) is a leading cause of disease burden in young children [1] and is associated with a range of poor psychological outcomes [2]. This includes post-injury behaviour changes [3], which can include excessive externalising behaviour, where the child demonstrates aggression, non-compliance, and irritability [4, 5]. The child may also exhibit poor self-regulation [6] and behave inappropriately in social settings [7, 8]. Internalising behaviours may also be present, in which the child is withdrawn and anxious [3], and parents may report personality changes [9].

The prevalence of such changes have been documented in 36% of severe TBI cases and 22% of moderate TBI cases [10]. Research also suggests that young children who experience a mild TBI requiring inpatient care are more likely to have behaviour problems than their uninjured peers [11]. Indeed, Catroppa and colleagues [12] showed that behaviour problems can emerge regardless of injury severity. These changes have been demonstrated to emerge early post-injury and increase over time [10] and may persist in the long-term [10, 11]. Behaviour disturbances post-injury have the potential to set individuals on a poor long-term trajectory, which may include difficulties in the classroom [10], anti-social behaviour [4], and increased risk of criminality [13]. Thus, problematic behaviour post-TBI is an outcome which requires attention and cannot be assumed to resolve without intervention.

### Parent outcomes when caring for a child post-TBI

Parental stress and family burden are also elevated following a child TBI. More specifically, behaviour disturbances post-TBI have been demonstrated to relate to higher parent distress and greater injury-related family burden [10]. Evidence suggests that this relationship is bi-directional, where parental distress, and mental health more generally, also uniquely predicts child behaviour problems [14]. Parents exhibiting greater distress are suggested to be less likely to adopt positive parenting styles to manage their child’s behaviour [15]. Relatedly, poorer parental communication [16] and disciplinary practices [17] relate to poorer post-TBI outcomes. Therefore, interventions which aim to reduce parent distress, and improve parental disciplinary styles and family functioning, may be particularly beneficial in improving post-paediatric TBI behaviour.

### The Signposts for Building Better Behaviour programme

‘Signposts for Building Better Behaviour’ (Signposts) is an intervention programme initially developed to assist parents in managing challenging behaviours in children with intellectual disability [18], with the additional aim of decreasing parent distress and increasing their sense of competence in managing their child’s behaviour. Signposts is a manualised programme in which parents read modules, watch videos, and complete homework exercises. Drawing on principles from behavioural therapies such as positive behaviour support, the programme aims to teach parents to conduct a functional analysis of a target behaviour, then design and implement an intervention to replace this behaviour with more desirable behaviour. The role of the Signposts therapist is to reiterate key messages from the module, answer any questions that the parent may have, and troubleshoot homework difficulties. The programme was designed to be delivered in a variety of modes of delivery, including a group format, as well as with clinician support via telephone, or in a self-directed manner (i.e. without the support of a clinician). However, research has suggested that those who receive support from a clinician during the programme are more likely to successfully complete the full programme [19].

Signposts has more recently been adapted to be relevant for parents of children with a TBI, with the inclusion of an additional paediatric TBI psycho-education module [20]. The modified programme was found to have high levels of consumer satisfaction for the parents of children with a TBI, who found the modules and the skills taught to be relevant and useful [21]. Parents reported significant reductions in challenging child behaviours, as well as significant reductions in dysfunctional parenting practices, stress, and family burden, when completing the programme through both group support and telephone support practices [22]. Evidence suggests good maintenance of these effects in the long-term post-intervention [23].

### The use of technology in delivering health interventions

Electronic health services, or ‘eHealth’, commonly refer to the ‘use of information technology in the delivery of health care’ [24]. Several studies report efficacy in the use of eHealth as a medium for delivering post-TBI behaviour interventions to families, including research utilising psycho-education and web-based family-oriented therapy, with associated improvements in caregiver distress [25–27].

The use of the Internet to deliver therapy for post-paediatric TBI behavioural disturbance has many benefits. Videoconferencing may be beneficial as parents would not require transport or childcare and could potentially avoid time taken from work to attend face-to-face therapy sessions [28]. Paediatric TBI causes a considerable degree of financial strain for families [29], and any method for alleviating this strain is worth investigating. Furthermore, the risk of sustaining a paediatric TBI is increased for those who live in remote areas [30], highlighting the need to increase access to interventions for those who live far from rehabilitative services.

While receiving telephone support for post-TBI rehabilitation can assist in removing some of these socioeconomic and geographical barriers, videoconferencing has several additional benefits [31]. Video technology means improved ability to detect non-verbal cues and provides an opportunity for including more than just one parent in the session. It enables shared viewing of programme materials such as videos, graphics, and homework exercises—none of which is possible via a traditional telephone. While videoconferencing is relatively new to post-paediatric TBI care, research in the related area of mental health intervention reports that videoconferencing has a comparable level of efficacy when compared to standard face-to-face delivery. For example, one randomised controlled trial comparing therapy across these modes of delivery found that post-therapy, 74.3% of patients treated via videoconferencing showed a reliable improvement in symptoms, compared to 75% when treated face to face [32]. Further, researchers have found there to be no significant difference between the two modes of delivery regarding the quality of the therapeutic alliance when rated by the client (*p* = 0.53) or the therapist (*p* = 0.60), or regarding client ratings of service satisfaction (*p* = 0.77) [33]. Several studies have reported that videoconferencing is more cost effective than standard face-to-face care delivery [34].

### Study aims

The primary aim of the current study is to investigate the feasibility and acceptability of conducting Signposts with videoconferencing as the mode of clinician support. In particular, this study intends to investigate this in families where the child sustained the TBI in early childhood, an age group that may be particularly prone to post-TBI poor outcomes [35–37] but, to date, has been relatively neglected with minimal attention directed to development of age-appropriate evidence-based interventions targeting behaviour problems.

More specifically, the study objectives are to:

1. Investigate the feasibility of delivering the programme with clinician support via videoconferencing, with the goal of developing a larger RCT. This will be investigated with the collection of data on sample retention rates, participant programme adherence, and duration to reach target sample size.
2. Collect information to inform on the clinical practicality of delivery through this medium, including the duration of videoconference sessions, rate of technology difficulties that cause disruption to sessions, and the time frame for programme completion (in weeks).
3. Explore the acceptability of the programme when delivered through this medium, with completion of a consumer satisfaction survey, as well as by enquiring on the participant’s comfort with using the technology. It is expected that, consistent with programme delivery through different modalities, the programme will have a high level of consumer satisfaction in this population when delivered with clinician support via videoconferencing.
4. Explore preliminary clinical outcomes across those who completed the programme compared with those on a wait-list, in order to inform likely efficacy estimates for a fully powered RCT. Primary measures will assess child behaviour, while secondary outcome measures will assess parent distress, parenting disciplinary style, parenting sense of competency, and family functioning. These will be measured using questionnaires considered standard for research involving the Signposts programme (see, for example, Woods and colleagues [23]). It is expected that those who complete Signposts will report reductions in post-TBI child behaviour problems, parental distress, and dysfunctional parenting, while increasing family functioning and parental sense of competency.

## Methods/design

### Overall study design

**Fig. 1:**
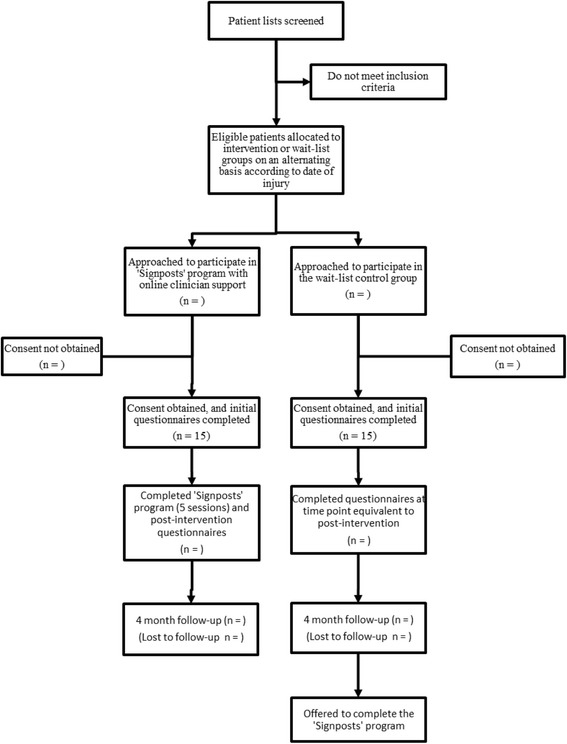
Participant flow diagram

The current pilot study employs a two-arm, parallel non-randomised design, in which a treatment as usual wait-list group is compared with a group in which the ‘Signposts’ programme is completed with clinician support via videoconferencing (see Fig. 1). The wait-list group may access any therapies or treatments considered standard treatment for a paediatric TBI.

Data will be collected at four time points: (i) pre-injury functioning at the time of enrolment in the study and then (ii) pre-intervention, (iii) immediately post-intervention, and (iv) at the 4-month follow-up post-intervention. Data collected from those who complete the intervention will be compared with data collected at equivalent time points from a wait-list control group. This study has been granted ethics approval by the Human Research Ethics Committee of the Royal Children’s Hospital, Melbourne.

### Participants

Participants are the primary caregivers (herein referred to as parents) of at least one child who has sustained a TBI within the prior 2 years. Children aged 2.0–6.11 at the time of injury, who sustained mild, moderate, or severe injuries, will be eligible to participate.

Inclusion criteria for the current study are that medical records suggest that parents and children have sufficient English skills, that the child has no prior history of neurological or developmental disorder, no diagnosed severe psychiatric disorder in the parent, and no documented evidence that the TBI was non-accidental.

In keeping with previous research [38], TBI will be classified by injury severity (mild, moderate, or severe). For young children, these categories are largely based on scores on the Paediatric Glasgow Coma Scale (GCS) [39], which provides ratings of the depth and/or duration of altered consciousness post-injury. As an adaptation to the initial scale devised for use in adults [40], GCS is instead rated in young children by recording behavioural rather than verbal responses. Scores are assigned on a scale of 3–15, where 3 indicates no verbal, motor, or visual responsiveness to stimuli, and 15 indicates full responsiveness.

Classifications of injury severity are made according to the following criteria:

(i) Mild: GCS 13–15, no evidence of mass lesion on CT/MRI and no neurological deficits
(ii) Moderate: GCS 9–12, mass lesion or other evidence of specific injury on CT/MRI and/or neurological impairment
(iii) Severe: GCS 3–8, mass lesion or other evidence of specific injury on CT/MRI and/or neurological impairment

### Measures

#### Injury and demographic variables

Injury and medical characteristics of the injured child are obtained, including lowest GCS, length of coma, neuroimaging abnormalities, injury aetiology, family structure, ethnicity, parental education, and parental occupation. Social adversity is calculated based on the Social Risk Index (SRI), which is comprised of six aspects of social status and provides an aggregate score, where a score of 0 or 1 denotes low social adversity, and a score of >2 denotes high social adversity [41].

#### Child behavioural outcomes

The pre-morbid, pre-intervention, and post-intervention behavioural functioning of the children is determined using the Child Behavior Checklist (CBCL) [42]. Two versions of the CBCL will be used. The preschool version of the CBCL is a standardised 100-item questionnaire, which was designed for use on children aged 1.5–5, while the school-aged version is a 113-item questionnaire designed for use on children aged 6–18. The age-appropriate version will be completed by the caregiver regarding their child’s behaviour. The current study used the three summary scores for analysis (total, internalising and externalising behaviour scales). *T* scores are derived for these scales (*M* = 50, SD = 10), where a *T* score greater than or equal to 60 denotes behaviour falling in the clinically significant range.

#### Family outcomes

Family functioning will be assessed with the self-report McMaster Family Assessment Device (FAD) [43, 44]. The 12-item summary scale, FAD-General Functioning (FAD-GF), will be used for analysis. High family dysfunction is indicated by a mean FAD-GF score >2.17 [45].

#### Parental functioning

Parental psychological distress will be measured using the Depression Anxiety Stress Scales (DASS) [46]. The DASS is a 42-item self-report inventory designed to measure the negative emotional states of depression, anxiety, and stress. Symptom levels on each subscale are categorised as falling in the normal, mild, moderate, severe, and extremely severe range according to the criteria outlined in Table 1.

**Table 1:** Subscale severity criteria for the DASS

Severity	Depression	Anxiety	Stress
Normal	0–9	0–7	0–14
Mild	10–13	8–9	15–18
Moderate	14–20	10–14	19–25
Severe	21–27	15–19	26–33
Extremely severe	28+	20+	34+

Lovibond and Lovibond [46]

The total for each subscale will be used for analysis in the current study.

The Parenting Scale (PS) will be used to measure different styles of disciplinary practices [47]. The PS is a 30-item questionnaire that yields three measures of dysfunctional disciplinary styles in parents: laxness, overreactivity, and verbosity. A total dysfunctional disciplinary style score can be calculated where lower scores reflect better parenting. This score will be used for analysis in the current study.

The Parenting Sense of Competence Scale (PSOC) will be used to measure the parent’s self-reported competency and satisfaction in their parenting [48]. The scale consists of 16 items, from which two dimensions can be derived, reflecting satisfaction with their parenting role and sense of self-efficacy in the parenting role. Total scores across these two dimensions will be used for analysis, with higher scores reflecting greater satisfaction and efficacy.

#### Programme satisfaction

Parent satisfaction with the Signposts programme, and its delivery, will be measured using the nine-item Consumer Satisfaction Scale (CSS), which was developed for the purpose of investigating satisfaction with the Signposts programme [18]. Items on this measure are recorded on a five-point Likert scale, in which participants indicate their level of agreement with a statement about their experience with the programme, from ‘Strongly Disagree’ to ‘Strongly Agree’.

### Procedure

#### Recruitment

Eligible parents are identified by clinicians working in relevant departments at the Royal Children’s Hospital and the Victorian Paediatric Rehabilitation Service in Melbourne. Parents will then be allocated to either the intervention or wait-list control group on an alternating basis according to date of injury.

Recruitment letters will be mailed to parents, introducing the study and inviting them to participate. Parents are provided with information statements and sign the consent form should they wish to participate. Parents who do not accept or decline participation within 2 weeks will be followed up with a phone call.

#### Data collection

Data will be recorded to capture the number of eligible parents approached, the number who consented to the programme, and the number who successfully completed the programme, as well as reasons for non-participation and non-completion.

Questionnaires will be administered using a secure online data collection instrument (RedCap), with parents sent web links at the appropriate time points. Upon enrolment in the study, parents will be sent the web-link questionnaires and asked to complete the outcome measure questionnaires retrospectively, reporting on their child and family’s functioning prior to the TBI. It is acknowledged that this may be vulnerable to parent reporting bias, particularly where children are several years post-injury. However, evidence suggests that post-TBI behavioural problems may not emerge or present for intervention until several years post-injury [10, 11], which means that including families several years post-injury more closely mirrors the clinical setting. It therefore seems reasonable to include such families in this feasibility study. Future research may aim to minimise this bias by reducing the eligible time since injury.

Prior to commencing the programme, approximately 1 month following initial questionnaire completion, parents will be asked to complete the measures again with regard to their family and child’s *current* functioning. Outcome questionnaires will again be sent in the form of web links via email immediately after the intervention and 4 months post-intervention. The approximate timeline for completion of these questionnaires is summarised in Table 2.

**Table 2:** Summary of measures used

Construct	Measure	Time point			
		1	2	3	4
Socioeconomic status	Social Risk Index	●			
Outcome measures					
Child behavioural outcomes	Child Behavior Checklist	●	●	●	●
Family outcomes	McMaster Family Assessment Device	●	●	●	●
Parental functioning					
Parental psychological distress	Depression Anxiety Stress Scales	●	●	●	●
Confidence and satisfaction in the parenting role	Parenting Sense of Competence Scale	●	●	●	●
Disciplinary practices	Parenting Scale	●	●	●	●
Programme satisfaction	Consumer Satisfaction Scale			●^a^	

Time point 1 = retrospectively completed about pre-injury behaviour, 2 = pre-intervention, 3 = post-intervention, 4 = 4 months post-intervention

^a^Intervention group only

#### Intervention procedure

Prior to commencement of this study, the Signposts clinician will complete the Professional Training Workshop through the Parenting Research Centre in Melbourne, where the original Signposts programme was developed. Workbooks and modules used in the current study are in the original hard copy form, available through the Parenting Research Centre. In addition, this project uses the stand-alone adjunct module ‘Dealing with a head injury in the family’, developed by Woods and colleagues [20].

Children must be at least 3 months post-injury before their parents commence the programme. When ready to commence the programme, participants engage in an initial face-to-face interview with the Signposts clinician, which provides the opportunity to build rapport and further explain the requirements of the programme, including demonstration of the iPads and the videoconferencing software. Initial modules required to begin the programme (1–3, 8, and 9) are provided at this session. The programme is commenced after this initial interview.

Parents work through module booklets in their own time and complete the relevant tasks in their workbook. On five occasions, approximately 2 weeks apart, parents engage in a one-on-one videoconference session with the Signposts clinician. The session follows the suggested structure for telephone support from the practitioner manual, which generally aims to support parents in completing homework and answer any questions or concerns that may arise.

In line with previous research [21], some programme modules were grouped together for logistical purposes. The approximate timeline for the completion of modules and videoconference sessions is presented in Table 3. Aside from the initial modules provided at the pre-intervention interview, subsequent modules (4–7) are individually sent to the family via post, as needed.

**Table 3:** Programme for completion of Signposts programme with online clinician support

Week	Parent homework	Clinician contact
0		Pre-intervention interview
1	Read modules 1–3, 8, and 9 Begin homework	
2	Complete homework for modules 1–3, 8, and 9 prior to videoconference	Videoconference #1 Homework reviewed for modules 1–3, 8, and 9
3	Read module 4 Begin homework	
4	Complete homework for module 4 prior to videoconference	Videoconference #2 Homework reviewed for module 4
5	Read module 5 Begin homework	
6	Complete homework for module 5 prior to videoconference	Videoconference #3 Homework reviewed for module 5
7	Read module 6 Begin homework	
8	Complete homework for module 6 prior to videoconference	Videoconference #4 Homework reviewed for module 6
9	Read module 7 Begin homework	
10	Complete homework for module 7 prior to videoconference	Videoconference #5 Homework reviewed for module 7
11	Complete post-intervention questionnaires Return iPad	

### Sample size

The current feasibility study sample will aim to consist of 15 parents who will participate in the intervention and a further 15 allocated to the wait-list control group. Studies also exploring the feasibility of family-centred behaviour interventions in this population used similar sample sizes [49–52] and detected promising preliminary results. With recruitment in this population frequently difficult and slow, we aim to measure the duration of time taken to recruit this sample in order to guide estimates of recruitment duration for a larger RCT.

### Data analysis

Frequencies and percentages will be used to describe the population across key demographic and injury variables. We will compare the intervention and wait-list control groups across key variables (age at injury, injury severity, social risk, pre-injury behaviour, and pre-intervention behaviour) to detect any significant difference which may later compromise the generalisation of the findings.

Investigating the feasibility, clinical practicality, and acceptability of Signposts with videoconferencing support will be exploratory in nature. The objective is to collect information on ease of use, participant satisfaction, homework completion rates, programme completion and adherence, and attrition rates. Rates of programme attrition will be reported, as well as consumer satisfaction with the programme (reported as mean scores and percentages for items on the CSS).

To examine preliminary data on the impact of the programme on key outcome areas, we will compare the post-intervention scores on the CBCL scales, FAD, DASS, PS, and PSOC across the intervention and control groups and will report the mean and standard deviation of each group across these measures. To report preliminary findings on the maintenance of any treatment effects, means and standard deviations will be reported to assess any change within the intervention group from post-intervention to 4-month follow-up across the CBCL scales, FAD, DASS, PS, and PSOC. These findings may inform efficacy estimates for a larger RCT.

## Discussion

This study is the first to investigate the feasibility and efficacy of a parenting training programme using videoconferencing as the support mode, for decreasing undesirable behaviours in children who sustained a TBI at an early age and improving parental post-TBI outcomes, such as mental health and sense of parenting efficacy. Preliminary findings of feasibility and efficacy of Signposts when delivered with an online support mode will pave the way for use of this method in a larger randomised controlled trial, with the aim of translation into clinical settings. For many families, when implemented clinically, online programme delivery would provide the opportunity to access services which would otherwise be costly and time consuming, particularly for those from more challenging socioeconomic backgrounds, or living in rural settings. This initial study also lays the foundation to further develop Signposts materials to be fully provided online.

## Abbreviations

CBCL: Child Behavior Checklist
CSS: Consumer Satisfaction Scale
DASS: Depression Anxiety Stress Scale
FAD: Family Assessment Device
FAD-GF: Family Assessment Device-General Functioning
GCS: Glasgow Coma Scale
PS: Parenting Scale
PSOC: Parenting Sense of Competence Scale
SRI: Social Risk Index
TBI: Traumatic brain injury
